# Conserved grasslands support similar pollinator diversity as pollinator-specific practice regardless of proximal cropland and pesticide exposure

**DOI:** 10.1098/rsos.231093

**Published:** 2023-11-22

**Authors:** Johanna M. Kraus, Kelly L. Smalling, Mark W. Vandever, Carrie E. Givens, Cassandra D. Smith, Dana W. Kolpin, Michelle L. Hladik

**Affiliations:** ^1^ Columbia Environmental Research Center, U.S. Geological Survey, Columbia, MO 65201, USA; ^2^ New Jersey Water Science Center, U.S. Geological Survey, Lawrenceville, NJ 08648, USA; ^3^ Fort Collins Science Center, U.S. Geological Survey, Fort Collins, CO 80526, USA; ^4^ Upper Midwest Water Science Center, U.S. Geological Survey, Lansing, MI 48911, USA; ^5^ Oregon Water Science Center, U.S. Geological Survey, Bend, OR 97701, USA; ^6^ Central Midwest Water Science Center, U.S. Geological Survey, Iowa City, IA 52240, USA; ^7^ California Water Science Center, U.S. Geological Survey, Sacramento, CA 95819, USA

**Keywords:** pollinator diversity, Conservation Research Program, wild bees, insecticides, flowering plant diversity, agriculture

## Abstract

Pollinator diversity and abundance are declining globally. Cropland agriculture and the corresponding use of agricultural pesticides may contribute to these declines, while increased pollinator habitat (flowering plants) can help mitigate them. Here we tested whether the relative effect of wildflower plantings on pollinator diversity and counts were modified by proportion of nearby agricultural land cover and pesticide exposure in 24 conserved grasslands in Iowa, USA. Compared with general grassland conservation practices, wildflower plantings led to only a 5% increase in pollinator diversity and no change in counts regardless of the proportion of cropland agriculture within a 1 km radius. Pollinator diversity increased earlier in the growing season and with per cent flower cover. Unexpectedly, neither insecticide nor total pesticide concentrations on above-ground passive samplers were related to pollinator diversity. However, pollinator community composition was most strongly related to date of sampling, total pesticide concentration, and forb or flower cover. Our results indicate very little difference in pollinator diversity between grassland conservation practices with and without wildflower plantings. Given the relatively high economic costs of wildflower plantings, our research provides initial evidence that investment in general grassland conservation may efficiently conserve pollinator diversity in temperate regions of intensive cropland agriculture.

## 1. Introduction

Insect pollinators are an integral part of natural and agricultural ecosystems [1,2]. Most flowering plants and economically important crops rely on insect pollinators for reproduction and seed production [3]. As a result, the recent precipitous decline in abundance and diversity of many insect pollinators may lead to dramatic ecological and economic disruptions [4–6]. For example, one-third of flowering plants would produce no seeds without insect pollinators, and crop production in the USA would be greatly decreased by a reduction in pollinators [2,3]. To predict and mitigate pollinator decline, there has been a groundswell of research into its causes [5–8], effects [1,2,9,10] and possible recovery [11–14]. Land-use intensification and use of pesticides, in particular, have been implicated as causes of decline [6,7,15], while increasing the availability and diversity of flowering plants (i.e. pollinator habitat) have been shown to help restore pollinator abundance and diversity [11,14]. Although these causes and solutions to declines receive much attention, less is known about how proximal agricultural land use and pesticide exposure may alter the effectiveness of pollinator habitat enhancement (i.e. wildflower plantings) aimed at conserving pollinator diversity, especially compared with the potential positive effects of other non-pollinator conservation practices.

Cropland agriculture (i.e. arable land) has been linked to both declines in flying insects and increased insect exposures to pesticides [5,15,16]. A study conducted concurrently with the present study found that concentrations and number of pesticides detected with above-ground passive samplers within conservation grasslands increased with proportion of cropland proximal to those grasslands (radius 1 km), although it found no relationship between proportion of proximal cropland and pesticide residues (number or concentration) in bee tissues at these sites [17]. In addition, the number of pesticides detected in the tissues of flying insects collected in nature conservation areas in Germany was related to the proportion of agricultural production area in a 2 km radius around the reserves [15]. Pesticides commonly applied to croplands, such as neonicotinoid insecticides and fungicides, can cause a variety of sublethal and lethal effects to pollinators including reduction in density, decreased foraging success, impaired development, and increased susceptibility to disease and parasites [5,18,19]. Because these compounds can reach nearby pollinator habitat through overspray, aerial drift and surface water flow, they also have the potential to reduce diversity and abundance of pollinators in conserved lands [11].

Conservation efforts aimed at increasing pollinator diversity commonly include creating pollinator-friendly habitat by growing flowering plants to support a diversity of pollinator species within agricultural landscapes [11,20]. In temperate regions, cropland margins are often used to provide additional pollinator habitat in otherwise highly modified landscapes [11,20,21]. In the USA, such efforts have occurred at the federal level since the Food, Conservation and Energy Act of 2008 [22] introduced language recognizing the importance of pollinators and allowed for measures to target the conservation of pollinator habitat. These measures include the US Department of Agriculture's (USDA) Conservation Reserve Program (CRP) Pollinator Habitat Conservation Practice (CP42), which involves planting a diverse mix of pollinator-friendly wildflowers (also called ‘forbs') in plots at least 0.5 acres in area (0.2 ha) [23]. The average cost of installing pollinator habitat is greater than CRP grassland practices; for example, USDA reports that as of 2015, average CP42 establishment cost was $248 per acre nationally (range: $49–$900 per acre), compared with $68–$85 per acre for grassland practices CP1 and CP2 [24]. Recent research has shown that CRP fields supplemented with wildflowers (CP42) showed inconsistent differences in number of bee taxa and bee abundance compared with those with a conventional grass mix [25], although wild bee abundance was 30–60% higher in CP42 fields compared with non-CRP fields or roadsides (i.e. increase from mean of 0.5 to 0.8 bees per transect) [26]. Little is known about the benefits of specific plantings for pollinators across a gradient of cropland agriculture and compared with the other less costly options supported by USDA [24] such as grassland conservation practices.

**Figure 1.**
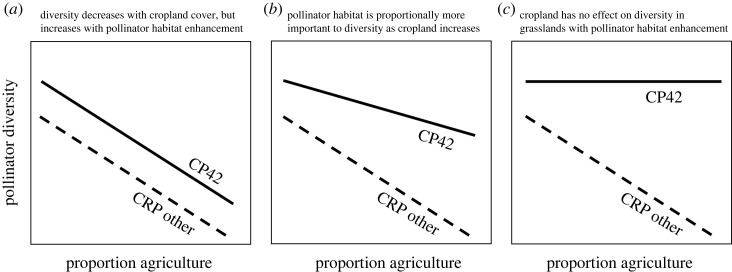
Hypothetical relationships between pollinator diversity surveyed in conserved grasslands enrolled in the U.S. Department of Agriculture (USDA) Conservation Reserve Program (CRP), and nearby cropland agriculture. Pollinator diversity is higher within pollinator habitat enhancement conservation practice (CP42) fields but decreases with cropland cover (*a*); the same is true as in (*a*), but the effects of nearby cropland on diversity are proportionately less in CP42 (*b*); the same is true as in (*b*), but the ameliorative effects of CP42 on diversity completely compensate for any negative effects of cropland agriculture (*c*).

The goal of our study was to determine the effectiveness of conservation practices implemented in promoting pollinator diversity across an agricultural gradient. Specifically, we were interested in testing whether pollinator habitat enhancement increased pollinator diversity and abundance compared with similar non-enhanced grasslands and whether this effect depended on proportion of nearby landscape being used for cropland agriculture. We compared pollinator habitat enhancement (CP42) and other conservation grasslands (CRP-other) across a gradient of cropland landcover in the same system to test the hypotheses that: (i) CP42 plantings would be related to substantial gains in pollinator diversity and abundance compared with CRP-other grasslands (figure 1*a*); (ii) increases in proximal cropland agriculture would be related to reductions in pollinator diversity and abundance in conserved grasslands with and without pollinator-specific plantings (CP42 and CRP-other, respectively) (figure 1*a*); and (iii) the ameliorative effect of CP42 plantings on pollinator diversity and abundance would be proportionately larger in conserved grasslands surrounded by cropland agriculture (compared with those surrounded by more natural habitat) (figure 1*b,c*). We further hypothesized that the mechanisms driving the effects of pollinator habitat enhancement and cropland cover on pollinators were: (i) increased diversity and cover of pollinator-friendly plants in CP42s and (ii) increased total pesticide concentrations (including herbicides, fungicides, and insecticides) in the environment as the amount of cropland progressively increased.

## 2. Methods

### 2.1. Site information

This work is part of a larger study of the effects of conservation practices and cropland agriculture on pollinator health in an intensively farmed region. Thus, site selection and study design has been previously described in Hladik *et al.* [17]. Briefly, 24 conservation grassland fields located in eastern Iowa, USA, the heart of the US Corn Belt and historic Great Plains ecosystem and a region with high density of CRP grassland fields and CP42 enrolment, were selected for study across a gradient of nearby cropland agriculture (electronic supplementary material, figure S.1) [17,27]. This region has a long agricultural history: for example, between 1833 and 1934, all the natural upland vegetation (i.e. prairie) and 66% of the original marshlands were converted to agriculture [28]. Fields selected in our study were at least 1 km apart to increase independence of pollinator responses among sites, were enrolled in the CRP for at least 3 years (planted before 2016) and were a minimum of 1 ha (0.01 km^2^). The 24 fields were a mix of CRP grasslands specifically planted with diverse (greater than nine species) pollinator-friendly forbs that bloom throughout the growing season (CP42, *n* = 13) and other CRP grassland fields (*n* = 11, practices included CP1, CP2, CP4D, CP10, CP25 and CP38) [27]. These other CRP grassland practices meet a variety of conservation needs and include some forb planting (from zero to a minimum of five species depending on the practice) but do not specifically target the needs of pollinators. The proportion of cropland agriculture (mainly maize and soya bean) proximal to the fields in a 1 km radius ranged from 6 to 85% [17,27]. Within each field, an approximately 1 ha plot (0.7–1.1 ha) was delineated near the centre of the field. All fields were sampled during two periods, 9–16 July and 13–19 August 2019, to capture pollinators, forb diversity and flowering early and late in the growing season.

### 2.2. Pollinator sampling

Plots were divided into equal quadrants (50 × 50 m) and sampled using sweep netting and beat sheeting [29,30]. Sweep netting targeted bees and flying insects around flowering plants, while beat sheeting targeted insects settled on flowering plants, especially beetles. When flowering plants were not present, sampling targeted insects near dominant vegetation (usually grasses). Sampling was conducted for 15 min, not including handling time, between 10.00 and 16.00 local time in each quadrant for a total of 1 person-hour per sampling type per plot. On a small number of occasions, sites were sampled for a few extra minutes (16 of 48 site dates for 7 ± 5 person minutes; mean ± standard deviation (s.d.)). During rain events, sampling was halted or postponed. All insects collected were placed in clean bags, preserved on dry ice in the field, and stored in a conventional freezer that same day.

In the laboratory, samples were sorted for identification. Bees (Insecta, Apoidea) were measured and photographed using an AmScope microscope fitted with an MU1400 digital camera, and intertegular distance (ITD; Cane [31]) was measured. Most bees were identified morphologically to genus or species using images of the bees, expert advice and keys such as DiscoverLife [32]. Genetic analysis of bee tissues was used to resolve some morphologically indistinguishable genus. All other insects were identified to the lowest taxonomic unit (usually genus) at the C.P. Gillette Museum of Arthropod Diversity at Colorado State University.

### 2.3. Vegetation sampling

A vegetation survey was conducted during each site visit. A quadrat (0.5 m^2^ PVC frame) was haphazardly placed in four locations in each quadrant, roughly along the diagonal line between the corner and the centroid of the hectare plot. In total, 16 quadrats were assessed in each hectare plot at each site visit. Per cent cover was estimated for total forb, total flowering head, total grass, total bare ground and total woody vegetation. In addition to the per cent cover of each category, forbs within each quadrat were further subdivided by species, per cent cover of the species and per cent cover of the flowering heads of that species. A list of blooming species was created for each site, even if the blooming forb was not present in one of the quadrats.

### 2.4. Pesticide metrics

To assess pollinator exposure to pesticides, silicone wristbands were deployed around the perimeter of each conserved grassland field for approximately 30 days prior to each pollinator sampling (see Hladik *et al*. [17] for more details). The bands were analysed for 180 pesticides and pesticide transformation products, and total pesticide and insecticide concentrations were calculated [17,27]. Pesticide concentrations as reported here are the summed concentrations of all agricultural pesticides (insecticides, fungicides and herbicides) detected per band per field. Insecticide concentrations are the summed concentrations of just the detected insecticides per band per field. We previously showed that there was a positive relationship between proportion of agriculture proximal to conserved grassland fields and pesticide concentrations accumulated on silicone bands at a site (a.k.a., ‘pesticide exposure') [17]. Pesticide exposure did not differ between CRP-other and CP42 fields [17]. Summed (total) pesticide concentration and summed insecticide concentration at a site were not significantly correlated due to the relatively low concentrations of insecticides compared with other compounds such as herbicides (see §2.6).

### 2.5. Biodiversity metrics

To reduce the likelihood of including non-pollinator phytophagous insect taxa associated with vegetation in the pollinator diversity metrics, pollinator diversity was calculated using adult taxa from the orders Hymenoptera, Coleoptera, Diptera and Lepidoptera only. These insects are referred to as ‘pollinators' for the remainder of the paper. For plants and insects, Shannon diversity indices were calculated using number of taxa (lowest taxonomic resolution for pollinators and plants). Pollinator counts and proportion plant cover were used as abundance estimates. The Shannon index is employed to facilitate comparison with other pollinator and CP42 publications. However, the Shannon index has been found to be insensitive or to show misleading responses to community changes [33]; to account for these problems, as recommended, we also present separate metrics of taxa composition (Bray–Curtis distance measures), abundance (pollinator counts or per cent plant cover), indicator taxa presence (indicator species analysis; §2.6) and taxa richness [33]. Taxa richness showed patterns intermediate to Shannon diversity and abundance for plants and insects; thus, taxa richness results are reported in tables but are not further discussed here.

### 2.6. Statistical analysis

Our statistical approach had two parts. First, ‘main effects' models were used to test the effects of land-use variables and sampling date (i.e. factors incorporated into the study design) on pollinator diversity, pollinator count, pollinator community composition, forb diversity and forb per cent cover. Second, ‘mechanistic' models were used to test the effects of factors potentially driving effects of land use and sampling date including forb diversity, forb cover, flowering plant cover, total pesticide concentration (the sum of herbicide, fungicide and insecticide concentrations) and insecticide concentration (sum of only insecticides). Statistical analyses were performed in R statistical software [34]. Graphs were produced in the ggplot package [35].

For the ‘main effects’ models, general linear models (GLMs) (‘glm' function in rms package) [36] or rank-based regression (when parametric assumptions were not met; ‘rfit' function in Rfit package) [37] were used to test the effects of pollinator habitat enhancement, proportion of proximal cropland agriculture, sampling date, and the interaction between pollinator habitat enhancement and proportion of proximal cropland agriculture on pollinator and forb diversity and abundance metrics in conserved grasslands. Multivariate analysis of variance using Bray–Curtis distance matrices (‘adonis' function in vegan package) [38] was used to separately test the main and interactive effects of pollinator habitat enhancement, proportion of proximal cropland agriculture, and sampling date on pollinator community similarity. Indicator species analyses (a.k.a., multilevel pattern analysis or ‘multipatt' function in indicspecies package) [39] were also used to identify pollinator species that were statistically more abundant in CP42 compared with other CRP grassland fields, and in July compared with August samples.

For the ‘mechanistic’ models, multiple regression using linear models (‘lm' function in stats package) [34] or rank-based regression were used to test the effects of forb diversity, forb cover, flowering plant cover, total pesticide concentration (sum of all detected pesticides) and insecticide concentration (sum of all detected insecticides) on pollinator diversity, richness and counts. Sampling date was included as an independent effect in this model to account for the effect of time on pesticide concentrations and forb diversity, such that spatial variation in the mechanistic factors could be linked to pollinator endpoints. Best-fit model for the multiple regression was chosen using bi-directional step-wise selection based on Akaike information criterion (AIC) (‘stepAIC' function in MASS package) [40] after checking for collinearity and linear relationships between variables. Dependent and independent variables were log- or square root-transformed to meet assumptions of linearity. No variables were removed due to collinearity. Multivariate analysis of variance (MANOVA) using Bray–Curtis distance matrices (‘adonis' function in vegan package) [38] was used to test the mechanistic effects of forb diversity, forb cover, flowering plant cover, total pesticide concentration and insecticide concentration on pollinator community similarity.

Non-metric multi-dimensional scaling (NMDS) and permutation test (‘envfit' function in vegan package) [38] were used to visualize Bray–Curtis distance measures and fit the effects of all independent variables including ‘main effects' and mechanistic factors (i.e. factors: pollinator habitat enhancement and sampling date; vectors: proportion cropland, forb diversity, forb cover, flowering plant cover, total pesticide concentration and insecticide concentration) onto an ordination using a permutation test.

To address some of the issues associated with assigning statistical significance based on arbitrarily assigned values (usually *p* < 0.05), results are discussed using the language of evidence as recommended by Muff *et al*. [41]. Thus, approximate values of *p* > 0.1 provide little to no evidence of a relationship between dependent and independent variables, 0.1 > *p* > 0.05 provides weak evidence, *p* < 0.05 provides moderate evidence, *p* < 0.01 provides strong evidence, and *p* < 0.001 provides very strong evidence.

## 3. Results

### 3.1. Pollinator habitat enhancement, cropland and date of sampling

**Figure 2.**
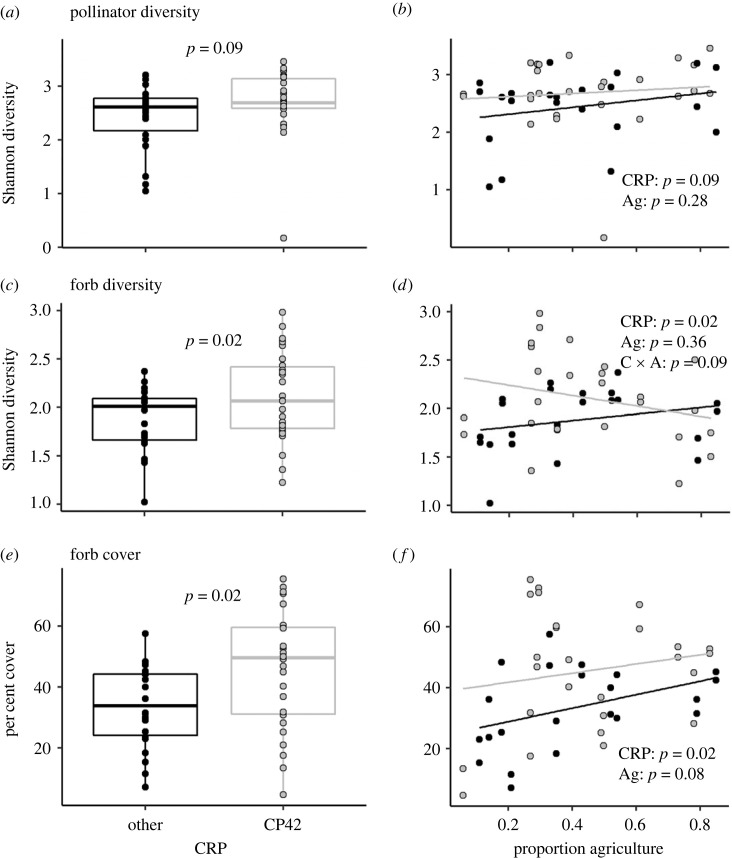
Actual relationships between pollinator and plant endpoints surveyed in conserved grasslands enrolled in the Conservation Reserve Program (CRP), and nearby cropland agriculture (Ag). Pollinator diversity (*a,b*), forb diversity (*c,d*), and forb cover (*e,f*) in CRP grassland fields with and without pollinator habitat enhancement (‘CP42' and ‘other', respectively) across a gradient of proportion of proximal cropland agriculture. Box plots represent the 95th, 75th, 50th (median), 25th and 5th percentiles of the data. C × A: interaction of CRP and Ag.

**Figure 3.**
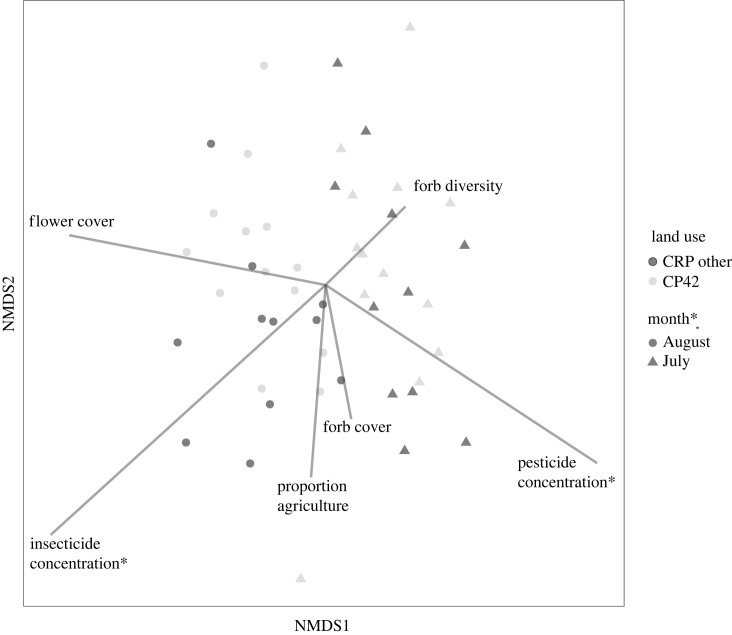
Two-dimensional non-metric multi-dimensional scaling (NMDS) ordination using Bray–Curtis distance measures fitted with environmental vectors (grey lines) and factors (data points at centroid). Vectors and factors were determined using 999 permutations and significance indicated (*) when *p* < 0.05. The lengths of lines are scaled by correlation (square root of *r*^2^, or goodness of fit statistic) to the strength of predictors (vectors). Colour of plotted points distinguishes community composition of Conservation Reserve Program (CRP) grassland fields with and without pollinator habitat enhancement (‘CP42' in grey and ‘other’ in black) and shape distinguishes between August (circle) and July (triangle) sampling period.

Over the duration of this study, we sampled 13 024 pollinators from 375 taxa. Results provided weak evidence that pollinator diversity was on average 0.15 units (approx. 5%) higher in CRP grassland fields that contained fields enrolled in the pollinator habitat enhancement practice (CP42) compared with CRP grassland fields without pollinator habitat enhancement (other) (figure 2 and table 1; electronic supplementary material, table S.1) [42]. However, there was little to no evidence that the pollinator communities differed by CRP practice (figure 3 and table 2; electronic supplementary material, table S.2): pollinator community composition did not differ and there was moderate evidence of differences for only 3 of 373 taxa (less than 1%) between CP42 and other CRP grassland practices. Results provided strong evidence suggesting that mean herbaceous flowering plant (forb) diversity was 0.2 units (approx. 9%) higher and per cent forb cover was 11 units (approx. 32%) higher in CP42 fields compared with other CRP grassland field.

**Table 1.** Statistical models testing effects of U.S. Department of Agriculture Conservation Reserve Program (CRP) practice, proportion of proximal cropland agriculture, sampling date, pesticide concentrations and forb diversity on insect and plant endpoints in July and August 2019 within managed grasslands (Iowa, USA). General linear models (GLM) and non-parametric rank-based regression (NPR) were used as indicated. ‘Mechanistic’ models represent best fit using AIC. Bold text indicates significant *p-*values (*p* < 0.05). Italics indicate marginally significant *p*-values (*p* < 0.1). For NPR, model test of significance is the reduction in dispersion from the null to the full model (*χ*^2^); the proportion variance explained by the model (*r*^2^) is multiple-robust *r*^2^ [37]. For GLM, model significance (versus null) was calculated using log-likelihood test. Differences in degrees of freedom (d.f._diff_) and log-likelihoods (*LL*_diff_) were used to calculate the test statistic (*χ*^2^), and *r*^2^ is the Cox–Snell maximum-likelihood pseudo-*r*^2^. Ag is the proportion nearby cropland agriculture.

model	d.f._diff_	*LL*_diff_	*χ^2^*	*P*	*r^2^*	type
**linear models – ‘main effects’**						
pollinator diversity ∼ CRP + agriculture + date	—	—	8.34	**<0**.**001**	0.36	NPR
pollinator richness ∼ CRP + agriculture + date	—	—	2.74	*0*.*055*	0.16	NPR
pollinator counts ∼ CRP + agriculture + date	−3	−3.52	6.88	*0*.*076*	0.13	GLM
forb diversity ∼ CRP + agriculture + date + CRP * Ag^a^	−4	−3.74	7.48	0.11	0.14	GLM
forb richness ∼ CRP + agriculture + date + CRP * Ag	—	—	3.62	**0**.**012**	0.25	
forb cover ∼ CRP + agriculture + date + Ag * date	−3	−5.46	10.9	**0**.**012**	0.20	GLM
**multiple regression – mechanistic**						
pollinator diversity ∼ date + pesticide concentration + flower cover	—	—	8.97	**<0**.**001**	0.38	NPR
pollinator richness ∼ date + insecticide concentration + forb diversity + forb cover + flower cover	−5	−13.6	27.2	**<0**.**001**	0.43	GLM
pollinator counts ∼ date + insecticide concentration + forb cover + flower cover	−4	−9.39	18.8	**<0**.**001**	0.32	GLM

^a^Interaction included when marginally significant.

**Table 2.** Permutation tests testing fit of variables affecting pollinator community composition (environmental vectors or factors) onto an NMDS ordination (envfit). The projections of points onto vectors have maximum correlation with corresponding environmental variables, and the factors show the averages of factor levels. The *r^2^* value indicates goodness of fit. For continuous variables this is equal to fitting a linear trend surface for a variable. Bold text indicates significant *p-*values (*p* < 0.05). Italics indicate marginally significant *p*-values (*p* < 0.1).

variable	NMDS1	NMDS2	*r*^2^	*p*
**vectors**				
proportion agriculture	−0.115	−0.993	0.026	0.54
forb diversity	0.840	0.542	0.015	0.74
forb cover (%)	0.280	−0.960	0.014	0.72
flower cover (%)	−0.992	0.127	0.109	*0*.*076*
pesticide concentration	0.918	−0.396	0.142	**0**.**037**
insecticide concentration	−0.859	−0.513	0.167	**0**.**009**
**factors**				
date	–	–	0.370	**0**.**001**
August	−0.265	0.030	–	–
July	0.265	0.030	–	**–**
CRP	–	–	0.021	0.370
other	0.010	−0.069	–	–
CP42	−0.008	0.058	–	**–**

There was no statistical evidence that pollinator diversity or community composition changed, and weak evidence that pollinator counts increased, with proportion of proximal cropland agriculture (figures 2 and 3, tables 1 and 2; electronic supplementary material, tables S.2 and S.3). There was no evidence that forb diversity changed with proportion of proximal cropland agriculture, but there was weak evidence that change in forb diversity over the agricultural gradient differed between fields with and without pollinator habitat enhancement (electronic supplementary material, table S.1). There was also weak evidence that forb cover increased slightly with proportion of proximal cropland agriculture (increase of 1.9% forb cover for every 10% increase in cropland agriculture; figure 2; electronic supplementary material, table S.1).

Results indicated strong evidence that pollinator diversity was 0.44 units higher during July sampling compared with August (table 1, electronic supplementary material, table S.1) and that pollinator community composition differed between sampling dates (figure 3 and table 2; electronic supplementary material, tables S.2 and S.3). There was moderate evidence for differences between sampling dates for 29 of 373 taxa (8%) (electronic supplementary material, table S.3).

### 3.2. Mechanisms driving effects on pollinator diversity, abundance and composition

**Figure 4.**
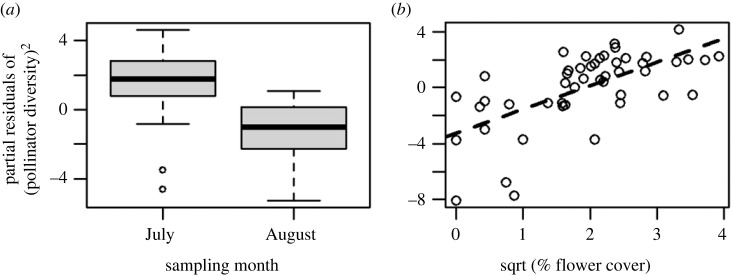
Results of multiple regression testing the effects of mechanistic factors on pollinator diversity in conserved grasslands. Partial residuals are plotted for factors retained in the best-fit model including sampling month (*a*), and flower cover (%) (*b*). Table 1 and electronic supplementary material, table S.1 for statistical results and parameter estimates. Box plots represent the 95th, 75th, 50th (median), 25th and 5th percentiles of the data.

Pollinator diversity at a site was associated with sampling date and flower cover (i.e. the two factors retained in the best-fit multiple regression model that showed strong evidence for a relationship; table 1; electronic supplementary material, table S.1, figure 4). These analyses estimated that pollinator diversity was 0.47 units higher in July than August and increased 1.0 unit over the range of flower cover (0–15.3) (figure 4 and table 1; electronic supplementary material, table S.1). Pollinator counts at a site were associated with insecticide concentrations and forb cover (i.e. the two factors retained in the best-fit multiple regression model that showed strong evidence of a relationship; table 1; electronic supplementary material, table S.1). Pollinator counts increased by 5.1-fold over the gradient in per cent forb cover (range: 4.6–76%) and (counterintuitively) increased by 6.7-fold over the gradient in insecticide concentrations (range: 5–1532 ng band^−1^; table 1; electronic supplementary material, figure S.2, table S.1). For the multivariate analyses, the MANOVA, which controlled for sampling date while testing other factors, provided moderate evidence that variance among pollinator community distance matrices was predicted by sampling date, total pesticide concentration and forb cover (electronic supplementary material, table S.2). The permutation tests on the NMDS provided moderate to weak evidence that community composition was related to sampling date, total pesticide concentration, insecticide concentration, and flower cover (figure 3 and table 2).

## 4. Discussion

As intended, the CP42 Pollinator Habitat Conservation Practice led to higher flowering plant diversity (9%) and forb coverage (32%) than in other CRP grassland practices. However, the gains for insects were not as large as for plants: pollinator diversity increased marginally by only 5%, and pollinator counts and community composition did not differ with pollinator habitat enhancement (less than 1% of taxa differed between CRP types). These findings were similar to other comparisons between CP42 and fields with grass seed mixes (i.e. CRP-other) in eastern Colorado. For example, Arathi *et al.* [25] found that abundance and number of bee genera were not consistently higher in CP42 fields; however, in that study, 40–60% of bee genera did not overlap between CP42 and CRP-other, indicating that diversity was enhanced by having both habitats, which was unlike our study. On the other hand, and perhaps not surprisingly, the difference in pollinator diversity we observed between CP42 and other CRP grassland practices (some of which do involve forb planting) is much smaller than the difference previously observed between CP42 and non-CRP fields and roadsides (30–60%) [26]. Thus, CRP grassland fields, but not non-CRP fields and roadsides, appear to provide sufficient pollinator habitat to support 95% of the diversity of pollinators supported by CP42 enhancements.

When measuring the contribution of pollinator habitat enhancements (CP42) on pollinator diversity, it is important to thoughtfully choose which habitats are being used for comparison. Here we chose to compare CP42 with other (less costly to implement) grassland conservation practices [24]. This choice strongly affected our results and realistically reflects the added benefit per dollar of CP42 to conservation agencies and farmers. CP42 establishment is relatively costly compared with other grassland practices, and despite the cost, there were 446 000 acres of CP42 plots by 2017 in the USA [43]. Other grassland practices may ultimately provide more benefit per dollar to pollinators, especially if more land can be enrolled because of the lower cost. However, further research would be needed on the effects of the CP42 practice in other regions (i.e. with different crop types and agricultural intensity), times of the growing season and planting management/success to test if the results we find here hold. Sampling date (early versus later in the growing season) will be an important factor to consider when comparing results, because of the strong effect sampling date had on pollinator diversity and community composition. Variation in management and planting success of individual CRP-other and CP42 fields are also likely to make a substantial difference for pollinator outcomes.

In contrast to the slightly positive effect of CP42 enhancement on pollinator diversity, proportion of cropland proximal to the grasslands (i) had no discernable effect on plant diversity, pollinator diversity, pollinator counts or pollinator community composition, although forb cover increased marginally, and (ii) did not increase the relative effect of the pollinator habitat enhancement on any pollinator endpoints. These findings contrast with both our expectations and some previous work. For example, the decline in biomass of flying insects in nature conservation areas in Germany was partially attributed to the effects of nearby arable land [15,16]. Furthermore, a global synthesis found that bee abundance and richness were higher in landscapes with more high-quality habitats, and bee richness benefited most from high-quality proximal land cover [44]. Finally, the efficacy of a European conservation program in increasing pollinator diversity depended in part on nearby land use: floral resources drove the response of pollinators to conservation practices, but the response to those resources was more positive in croplands (versus grasslands) located in simple landscapes [11]. It is possible that insect pollinators in an intensively farmed region like the State of Iowa, where 85% of the land surface is farmed [45] mostly as a monoculture of maize and soya bean, comprised taxa that are less sensitive to chemical exposures associated with croplands and thus more able to respond to local resource availability regardless of the proportion nearby cropland cover (similar to patterns observed in aquatic insect communities adjacent to agriculture) [46–48].

We hypothesized that the responses of pollinators to pollinator habitat enhancement and nearby cropland in conserved grasslands were probably associated with the effects of these land-use changes on resource availability (i.e. flowering plants) and pesticide exposure. The connection between land-use change on pollinators and the hypothesized mechanistic drivers of those effects appeared to hold for pollinator habitat enhancement but was less straightforward for cropland. For example, similar to many published accounts, the small increase in pollinator diversity promoted by CP42 found here was probably associated with the positive effect of flower cover and the changes in pollinator community composition that were linked to flower and forb cover [11,14]. On the other hand, our results indicated that pollinator diversity was not related to total pesticide or insecticide exposures; community composition was related to total pesticide and insecticide exposure; and pollinator counts counterintuitively increased with insecticide concentrations. This non-straightforward link between exposure to agricultural chemicals in conserved grasslands and pollinator responses is also not unprecedented. The effects of proximal chemical applications on pollinators in conservation grasslands were likely to be multi-factorial, with some factors like insecticides potentially causing negative effects at high concentrations and positive or hormetic responses when present at sublethal concentrations [5,49]. There is also the possibility that capture rates during sampling could be increased by the effects of some insecticides as a result of behavioural changes in the pollinators [50], although we cannot test this hypothesis with our data.

Given the global vulnerability of agricultural production owing to pollinator decline [9], the question of how to conserve pollinator diversity and abundance in agricultural regions has both economic and ecological relevance. Our area of study in the Midwestern USA has a history of intense farming. Within this context, present-day conservation areas such as managed grasslands provide critical habitat and resources for pollinators, increasing nutrient and soil retention as well as pollinator abundance and diversity [51–53]. However, questions about how to best manage conservation areas for pollinators, and the effects of proximal land use on their efficacy, still abound. Ultimately, our research (and others') suggests that flower and forb cover in managed grasslands improves pollinator diversity and abundance. However, we found very little difference in pollinator diversity between conservation practices compared in our study, suggesting that CRP grassland practices are as effective as CP42 for promoting pollinator diversity. From a conservation perspective, grassland refugia are needed to conserve pollinator diversity in altered ecosystems, but additional wildflower planting practices, taking into account the realized increase in floral resources and diversity, do not appear to derive additional large benefits. As pollinator decline will probably continue to be an issue for both ecological and agricultural ecosystems, identifying conservation management options that are optimized for both ecological and economic outcomes (i.e. sustainable and effective) will continue to be an important goal for pollinator research.

## Data Availability

Data used in this paper are publicly available at https://doi.org/10.5066/P9Q0NAF8 [42]. Supplementary material is available online [54].
